# Theory of Interacting Quantum Gases

**DOI:** 10.6028/jres.101.047

**Published:** 1996

**Authors:** H. T. C. Stoof, M. Bijlsma, M. Houbiers

**Affiliations:** University of Utrecht, Institute for Theoretical Physics, Princetonplein 5, P.O. Box 80.006, 3508 TA Utrecht, The Netherlands

**Keywords:** Bose-Einstein condensation, degenerate atomic gases, mechanical stability, superconductivity, superfluidity

## Abstract

We present a unified picture of the interaction effects in dilute atomic quantum gases. We consider fermionic as well as bosonic gases and, in particular, discuss for both forms of statistics the fundamental differences between a gas with effectively repulsive and a gas with effectively attractive inter atomic interactions, i.e., between a gas with either a positive or a negative scattering length.

## 1. Introduction

One of the most important objectives of atomic physics in the last two decades, has been the achievement of Bose-Einstein condensation in a weakly interacting gas. It is, therefore, not surprising that the observation of Bose-Einstein condensation in the magnetically trapped alkali vapors ^87^Rb [1] ^7^Li [2], and ^23^Na [3], is considered to be one of the most exciting events of the past year. This is not in the least the case, because now that the successful road towards Bose-Einstein condensation has been found, one can safely assume that gases of atomic ^85^Rb, ^133^Cs, and ^1^H will also be Bose condensed in the near future and that a large number of different experimental systems will be available to study various aspects of the condensation phenomenon in detail.

Moreover, both lithium and hydrogen have stable fermionic isotopes which can be trapped and cooled in a manner similar to that of their bosonic counterparts. For atomic ^6^Li this has already been achieved [4], but magnetically trapped deuterium (^2^H) has not been observed yet because the loading of the trap cannot be accomplished in the same way as in the case of atomic hydrogen. This is presumably caused by the fact that i) deuterium binds more strongly to a superfluid helium film, ii) the surface recombination rate is much larger, and iii) the sample is contaminated with atomic hydrogen [5]. Nevertheless, it is to be expected that these experimental difficulties can be overcome and that one will soon be able to study both these Fermion gases in the degenerate regime.

In view of the exciting developments mentioned above, it appears justified to present here an overview of the physical properties of all these weakly interacting quantum gases. We thereby intend to bring out most clearly the differences due to the Fermi or Bose statistics on the one hand, and due to the effective interatomic interaction being either repulsive or attractive on the other. This division in the focus of the paper is also reflected in its layout. In Sec. 2 we present a general introduction to the physics of the atomic quantum gases of interest. In particular, we specify the approximations that are allowed for these quantum systems, and subsequently show how one can determine the effective interaction between the atoms. Using the latter result, we consider in Sec. 3 the Fermi and in Sec. 4 the Bose gases. Because we wish to bring out the different physical properties most clearly, we treat there only the homogeneous case. The experimentally more realistic case of a gas in a harmonic oscillator potential, however, does not lead to any qualitative differences, except for a Bose gas with an effectively attractive interaction. The latter possibility is therefore briefly discussed in Sec. 5, where we point out some problems that remain for the future.

## 2. Interacting Quantum Gases

All atoms mentioned in the introduction are members of the first primary group of the Periodic Table of Elements and, therefore, have a magnetic moment ***μ*** that is equal to the sum of the magnetic moments of the electron spin ***s*** (with *s* = 1/2) and the nuclear spin ***i***. Due to this magnetic moment, the atoms can easily be trapped by an inhomogeneous magnetic field and can be evaporatively cooled by a resonant microwave field that flips the magnetic moment. The successful experiments of the past year have shown that this is a great advantage. However, the magnetic moment also gives rise to an important problem because two-body collisions in the gas can lead to a change in the component of the magnetic moment parallel to the magnetic field and thus to a decay of the atomic density in the magnetic trap.

This decay mechanism seriously limits the lifetime of the gas, and can in principle be caused both directly by the magnetic dipole-dipole interaction *V*^d^ and by the central (singlet/triplet) interaction *V*^c^ in combination with the hyperfine interaction *V*^hf^ = *a*_hf_***s*** · ***i*** [6]. In general, the typical time scale for the former process is much larger than that for the latter, and the gas spontaneously spin-polarizes to a state in which the lifetime *τ*_inel_ is determined solely by the magnetic dipole-dipole interaction. There are roughly speaking two scenarios: at small magnetic fields *B* << *a*_hf_/*μ*_e_, where *μ*_e_ denotes the electron magneton, the decay due to the central interaction can only be avoided by polarizing both the electron and the nuclear spin along the direction of the magnetic field, i.e., all the atoms are in the spin state |*m*_s_ = 1/2, *m_i_* = *i*〉. This corresponds to a so-called doubly spin-polarized atomic gas. However, for large magnetic fields *B* >> *a*_hf_/*μ*_e_, the nuclear spin does not necessarily have to be polarized to obtain a relatively long lifetime and one can essentially trap any mixture of the states |*m*_s_ = 1/2, *m_i_*〉 with an arbitrary projection of the nuclear spin. In this case we are dealing with an (electron) spin-polarized atomic gas.

It is important to note that although the central interaction does not determine the lifetime of the (doubly) spin-polarized gas, it does determine the typical timescale *τ*_el_ for elastic collisions. (See, however, Sec. 3 for one exception.) Because *τ*_el_ << *τ*_inel_ for not too low temperatures, we can consider the spatial degrees of freedom of the gas to be in equilibrium, whereas the spin degrees of freedom clearly are not. For time scales short compared to the lifetime, we are thus allowed to apply equilibrium statistical mechanics to the gas and our main problem is the accurate treatment of the elastic part of the central interaction, which for the (doubly) spin-polarized gases of interest is the triplet interaction *V*_T_.

The determination of the effect of the interaction on the thermodynamic properties of a quantum gas is considerably simplified by the presence of two small parameters in the problem. In terms of the density *n* of the gas, the range of the interaction *r*_V_, and the thermal de Broglie wavelength $\Lambda =\sqrt{2\pi \hbar^{2}/mk_{B}T}$ of an atom with mass *m*, the two small parameters are the gas parameter *nr*_V_^3^ and the quantum parameter *r*_V_/*Λ*. Physically, the condition *nr*_V_^3^ << 1 expresses the fact that we are dealing with a dilute system in which it is highly improbable that three particles are so close together that they can interact with each other simultaneously. As a result we can neglect three-body processes and only have to account for all two-body processes. Furthermore, the condition *r*_V_/*Λ* << 1 shows that if two atoms interact, their relative angular momentum can only be equal to zero. In combination, we therefore conclude that for an accurate description of a quantum gas we only need to consider all possible two-body *s*-wave scattering processes.

### 2.1 Two-Body Transition Matrix

To explain how this may be achieved, we first of all introduce the appropriate “grand-canonical” Hamiltonian of the (doubly) spin-polarized atomic gas. Using the language of second quantization [7], it reads

$$\begin{array}{c} H=\sum_{\alpha }\sum_{k}\left(\varepsilon_{k,\alpha }-\mu_{\alpha }\right)a_{k,\alpha }^{†}\;a_{k,\alpha }\\ +\frac{1}{2V}\sum_{\alpha \alpha \prime }\sum_{k\prime kq}V_{q}a_{k+q,\alpha }\;a_{k\prime -q,\alpha \prime }^{†}a_{k\prime ,\alpha \prime }a_{k,\alpha }\equiv H_{0}+H_{\mathrm{int}}, \end{array}$$ (1)

where $a_{k,\alpha }^{†}$ creates and *a*_k,_*_α_* annihilates an atom in the (electron and nuclear) spin state |*α*〉 and the momentum eigenstate |***k***〉 of a cubic box with volume *V* and periodic boundary conditions. As always, these creation and annihilation operators obey the (anti)commutation relations ${\left[a_{k,\alpha },\;a_{k\prime ,\alpha \prime }^{†}\right]}_{\pm }=\delta_{k,k\prime }\;\delta_{\alpha ,\alpha \prime }$ and ${\left[a_{k,\alpha },a_{k\prime ,\alpha \prime }\right]}_{\pm }={\left[a_{k,\alpha }^{†},a_{k\prime ,\alpha \prime }^{†}\right]}_{\pm }=0$ if the atoms are fermions or bosons, respectively. Moreover, *V_q_* = ∫d***x*** *V*_T_(***x***) e^−^*^i^^q^*^·^*^x^* denotes the Fourier transform of the triplet interaction and *ε*_*k*,*α*_ = *ħ*^2^***k***^2^/2*m* + *ε_α_* ≡ *ε_k_* + *ε_α_* is the energy of the single particle states |***k***, *α*〉. Note that both the spin state |*α*〉 and its energy *ε*_α_ depend on the applied magnetic field ***B*** due to the Zeeman interaction *V*^z^ = − ***μ*** · ***B***. For any particular atom this dependence can easily be determined by diagonalization of the spin part of the atomic Hamiltonian, i.e., of *V^hf^* + *V^z^*, but it is not explicitly calculated here since it plays only a minor role in the following. Note also that in agreement with our previous remarks, we have only assumed that the spatial degrees of freedom are in equilibrium and we have, therefore, introduced a different chemical potential *μ*_α_ for each spin state |*α*〉.

Next we consider the effect of the interaction *H*_int_ on an “initial” state |*i*〉 of two atoms. In particular, we take |*i*〉 to be the state in which the occupation numbers of all the single particle states are zero except for *N*_*K*/2+*k*,*α*_ and *N*_*K*/2−*k*,*α*__′_, which are equal to one. In lowest order in the interaction, the energy of this state is *E_i_* = *ε_K_*_/2+*k*,*α*_ + *ε_K_*_/2 −__*k*,*α*__′_ if we use that the chemical potentials for this two-body problem are equal to zero. Applying standard perturbation theory, we find that this energy is shifted due to the interaction by an amount

$$\begin{array}{c} \Delta E_{i}=\langle i\left|H_{\mathrm{int}}\right|i\rangle +\\ \sum_{f\neq i}\langle i\left|H_{\mathrm{int}}\right|f\rangle \frac{1}{E_{i}-E_{f}}\langle f\left|H_{\mathrm{int}}\right|i\rangle +\ldots , \end{array}$$ (2)

where the “final” state |*f*〉 is an eigenstate of *H*_0_ with energy *E_f_*. Moreover, due to the possibility of scattering to other momentum states, the state |*i*〉 also acquires a finite lifetime *τ_i_*, which up to second order perturbation theory is found from Fermi’s Golden Rule:

$$\frac{1}{\tau_{i}}=\frac{2\pi }{\hbar }\sum_{f}\delta \left(E_{i}-E_{f}\right){\left|\langle f\left|H_{\text{int}}\right|i\rangle \right|}^{2}.$$ (3)

Introducing the usual notation 1/(*x* + *i*0) for the limiting procedure lim*_h_*_↓0_ 1/(*x* + *iη*), both these results can be conveniently combined into

$$\begin{array}{c} \Delta E_{i}-\frac{i\hbar }{2\tau_{i}}=\langle i\left|H_{\mathrm{int}}\right|i\rangle +\sum_{f}\langle i\left|H_{\mathrm{int}}\right|f\rangle \\ \frac{1}{E_{i}-E_{f}+i0}\langle f\left|H_{\mathrm{int}}\right|i\rangle +\ldots . \end{array}$$ (4)

The relevant matrix elements of the interaction *H*_int_ are easily evaluated and we obtain that

$$\begin{array}{l} \Delta E_{i}-\frac{i\hbar }{2\tau_{i}}=\frac{1}{V}\{V_{0}+\frac{1}{V}\sum_{k\prime }V_{k-k\prime }\frac{1}{\hbar^{2}\left(k^{2}-{k\prime }^{2}\right)/m+i0}\\ V_{k\prime -k}+\ldots \mp \delta_{\alpha ,\alpha \prime }(V_{-2k}+\frac{1}{V}\sum_{k\prime }V_{-k-k\prime }\\ \frac{1}{\hbar^{2}\left(k^{2}-{k\prime }^{2}\right)/m+i0}V_{k\prime -k}+\ldots )\}, \end{array}$$ (5)

which in the continuum limit *V* → ∞ becomes

$$\begin{array}{c} \Delta E_{i}-\frac{i\hbar }{2\tau_{i}}=\frac{1}{V}\{V\left(0\right)+\int \frac{dk\prime }{{\left(2\pi \right)}^{3}}V\left(k-k\prime \right)\\ \frac{1}{\hbar^{2}\left(k^{2}-k^{\prime 2}\right)/m+i0}V\left(k\prime -k\right)+\ldots \\ \mp \delta_{\alpha ,\alpha \prime }(V\left(-2k\right)+\int \frac{dk\prime }{{\left(2\pi \right)}^{3}}V\left(-k-k\prime \right)\\ \frac{1}{\hbar^{2}\left(k^{2}-k^{\prime 2}\right)/m+i0}V\left(k\prime -k\right)+\ldots )\}. \end{array}$$ (6)

The higher order contributions can be calculated in a similar way and we actually find that the complete perturbation series can be summed byintroducing the so-called two-body T(ransition) matrix *T*^2^*^B^*(***k***′, ***k***; *E*), which obeys the famous Lippman-Schwinger equation [8]

$$\begin{array}{c} T^{2B}\left(k\prime ,k;E\right)=V\left(k\prime -k\right)+\int \frac{dk\prime \prime }{{\left(2\pi \right)}^{3}}V\left(k\prime -k\prime \prime \right)\\ \frac{1}{E-\hbar^{2}k^{\prime \prime 2}/m+i0}T^{2B}\left(k\prime \prime ,k;E\right) \end{array}$$ (7)

and is proportional to the amplitude for two atoms to collide with a kinetic energy *E* in the center-of-mass frame and change their relative momentum from *ħ****k*** to *ħ****k***′. Indeed, a comparison with Eq. (6) shows that the exact result is

$$\begin{array}{c} \Delta E_{i}-\frac{i\hbar }{2\tau_{i}}=\frac{1}{V}\{T^{2B}\left(k,k;\hbar^{2}k^{2}/m\right)\\ \mp \delta_{\alpha ,\alpha \prime }T^{2B}\left(-k,k;\hbar^{2}k^{2}/m\right)\}. \end{array}$$ (8)

We therefore conclude that for a system of two atoms we can account for all two-body processes by first replacing in the Hamiltonian of Eq. (1) the potential *V_q_* by the transition matrix *T*^2^*^B^*((***k*** − ***k***′)/2 + ***q***, (***k*** − ***k***′)/2; *ħ*^2^(***k*** − ***k***′)^2^/4*m*) and then using only first-order perturbation theory to avoid a double counting of the effects of the interaction.

### 2.2 Many-Body Transition Matrix

In a gas we are of course not dealing with only two atoms, but with a large number *N* = *nV* of atoms at a temperature *T* = 1/***k***_B_*β*. This implies that we should repeat our previous calculation for a grand canonical ensemble of “initial” states, in which the single particle states |***k***,*α*〉 have the average occupation numbers *N*_*k*,*α*_ = (exp *β* (*ε*_*k*,*α*_ − *μ_α_*) ± 1)^−1^ and the chemical potentials *μ*_α_ are determined from

$$N=\sum_{\alpha }\sum_{k}N_{k,\alpha }\equiv \sum_{\alpha }N_{\alpha }.$$ (9)

The average zeroth-order energy in this ensemble is equal to

$$E_{i}=\sum_{\alpha }\sum_{k}\left(\varepsilon_{k,\alpha }-\mu_{\alpha }\right)N_{k,\alpha }$$ (10)

and its average energy shift and lifetime due to the interaction *H*_int_ can now be calculated perturbatively by averaging Eq. (4). Performing this thermal average is somewhat cumbersome, but ultimately we find

$$\begin{array}{c} \Delta E_{i}-\frac{i\hbar }{2\tau_{i}}=\frac{1}{2V}\sum_{\alpha \alpha \prime }\sum_{\mathrm{Kk}}\;\\ N_{K/2+k,\alpha }N_{K/2-k,\alpha \prime }\times \{V_{0}+\frac{1}{V}\sum_{k\prime }\;\\ V_{k-k\prime }\frac{1\mp N_{K/2+k\prime ,\alpha }\mp N_{K/2-k\prime ,\alpha \prime }}{\hbar^{2}\left(k^{2}-k^{\prime 2}\right)/m+i0}V_{k\prime -k}+\ldots \\ \mp \delta_{\alpha ,\alpha \prime }(V_{-2k}+\frac{1}{V}\sum_{k\prime }\;\\ V_{-k-k\prime }\frac{1\mp N_{K/2+k\prime ,\alpha }\mp N_{K/2-k\prime ,\alpha \prime }}{\hbar^{2}\left(k^{2}-k^{\prime 2}\right)/m+i0}V_{k\prime -k}+\ldots )\}, \end{array}$$ (11)

neglecting between the curly brackets terms that are either of order *O*(*V_0_*(*nr_V_*^3^)^1/3^) or of order *O*(*V_0_r_V_*/*Λ*). (Diagrammatically speaking, we neglect the four “bubble” diagrams compared to the two “ladder” diagrams.)

The appearance of the factor 1 ∓ *N*_*K*/2 +_ *_k_*_′,_*_α_*_′_ ∓ *N*_*K*/2−_*_k_*_′,_*_α_*_′_ in this expression may appear surprising at first instance. It has, however, a clear physical meaning that becomes evident when we consider the contribution to the average lifetime *τ_i_* from a scattering process with *α* ≠ *α*′. Such a contribution is given by

$$\begin{array}{c} \frac{2\pi }{\hbar }\frac{1}{V}\sum_{k\prime }\delta \left(\frac{\hbar^{2}k^{2}}{m}-\frac{\hbar^{2}k^{\prime 2}}{m}\right){\left|V_{k-k\prime }\right|}^{2}N_{K/2+k,\alpha }N_{K/2-k,\alpha \prime }\\ \left(1\mp N_{K/2+k\prime ,\alpha }\mp N_{K/2-k\prime ,\alpha \prime }\right) \end{array}$$

and consists of the sum of a loss rate

$$\begin{array}{c} \frac{2\pi }{\hbar }\frac{1}{V}\sum_{k\prime }\delta \left(\frac{\hbar^{2}k^{2}}{m}-\frac{\hbar^{2}k^{\prime 2}}{m}\right)\\ {\left|V_{k-k\prime }\right|}^{2}N_{K/2+k,\alpha }N_{K/2-k,\alpha \prime }\\ \left(1\mp N_{K/2+k\prime ,\alpha }\right)\left(1\mp N_{K/2-k\prime ,\alpha \prime }\right) \end{array}$$

due to the collision of two particles, and a production rate

$$\begin{array}{c} -\frac{2\pi }{\hbar }\frac{1}{V}\sum_{k\prime }\delta \left(\frac{\hbar^{2}k^{2}}{m}-\frac{\hbar^{2}k^{\prime 2}}{m}\right){\left|V_{k-k\prime }\right|}^{2}\\ N_{K/2+k,\alpha }N_{K/2+k,\alpha \prime }N_{K/2+k\prime ,\alpha }N_{K/2+k\prime ,\alpha \prime } \end{array}$$

due to the collision of two holes. For a scattering process with *α* = *α*′ we can obtain a similar interpretation, although it is slightly more complicated in this case due to the interference effects.

Following our discussion of the two-atom problem in Sec. 2.1, we see that we can now include all two-body processes by introducing a many-body T(ransition) matrix $T_{\alpha ,\alpha \prime }^{MB}(k\prime ,k,K;E)$ which obeys

$$\begin{array}{c} T_{\alpha ,\alpha \prime }^{MB}(k\prime ,k,K;E)=V(k\prime -k)\\ +\int \frac{dk\prime \prime }{{\left(2\pi \right)}^{3}}V\left(k\prime -k\prime \prime \right)\frac{1\mp N_{\alpha }\left(K/2+k^{\prime \prime }\right)\mp N_{\alpha \prime }\left(K/2-k\prime \prime \right)}{E-\hbar^{2}k^{\prime \prime 2}/m+i0}\\ T_{\alpha ,\alpha \prime }^{MB}\left(k\prime \prime ,k,K;E\right) \end{array}$$ (12)

in the continuum limit. Using Eq. (7), this is equivalent to

$$\begin{array}{c} T_{\alpha ,\alpha \prime }^{MB}(k\prime ,k,K;E)=T^{2B}(k\prime ,k;E)\\ \mp \int \frac{dk\prime \prime }{{\left(2\pi \right)}^{3}}T^{2B}\left(k\prime ,k\prime \prime ;E\right)\\ \frac{N_{\alpha }\left(K/2+k\prime \prime \right)+N_{\alpha \prime }\left(K/2-k\prime \prime \right)}{E-\hbar^{2}k^{\prime \prime 2}/m+i0}T_{\alpha ,\alpha \prime }^{MB}(k\prime \prime ,k,K;E), \end{array}$$ (13)

which is more convenient for our purposes since it offers the opportunity to explore the consequences of the small parameters *nr_V_*^3^ and *r_V_*/*Λ*. Indeed, as a result of these small parameters the relevant momenta and energies are always small compared to *ħ*/*r_V_* and *ħ*^2^/*mr_V_*^2^, respectively, and we can safely neglect the momentum and energy dependence of the two-body *T* matrix. Consequently, Eq. (13) is immediately solved by

$$\begin{array}{c} T_{\alpha ,\alpha \prime }^{MB}\left(k\prime ,k,K;E\right)≃\\ \frac{T^{2B}\left(0,0;0\right)}{1\pm T^{2B}\left(0,0;0\right)\Xi_{\alpha ,\alpha \prime }\left(K;E\right)}, \end{array}$$ (14)

where the quantity *T*^2^*^B^*(***0***, ***0***; 0) = 4*πaħ*^2^/*m* is proportional to the (triplet) *s*-wave scattering length *a* and *Ξ*α,_α′_ (***K***; *E*) is defined by

$$\begin{array}{c} \Xi_{\alpha ,\alpha \prime }\left(K;E\right)\equiv \\ \int \frac{dk\prime \prime }{{\left(2\pi \right)}^{3}}\frac{N_{\alpha }\left(K/2+k\prime \prime \right)+N_{\alpha \prime }\left(K/2-k\prime \prime \right)}{E-\hbar^{2}k^{\prime \prime 2}/m+i0}. \end{array}$$ (15)

Moreover, using this solution we find that to a good approximation

$$\begin{array}{c} \Delta E_{i}-\frac{i\hbar }{2\tau_{i}}=\frac{1}{2V}\sum_{\alpha \alpha \prime }\sum_{\mathrm{Kk}}N_{K/2+k,\alpha }N_{K/2-k,\alpha \prime }\\ \left(1\mp \delta_{\alpha ,\alpha \prime }\right)T_{\alpha ,\alpha \prime }^{MB}\left(0,0,K;\hbar^{2}k^{2}/m\right), \end{array}$$ (16)

which can again be reproduced by first-order perturbation theory if we replaceV*_q_* in Eq. (1) by

$$T_{\alpha ,\alpha \prime }^{MB}\left(0,0,k+k\prime ;\hbar^{2}{\left(k-k\prime \right)}^{2}/4m\right).$$

With the last conclusion we have basically achieved our objective of summing all two-body *s*-wave scattering processes. In summary, we have shown that this requires the introduction of a many-body *T* matrix, which physically also incorporates the fact that the collisions of interest take place in a (gaseous) medium and are, therefore, influenced by the usual Fermi-blocking or Bose-enhancement factors [7]. As a result, the transition matrix $T_{\alpha ,\alpha }^{MB}(k\prime ,k,K;E)$ acquires a dependence on the center-of-mass momentum *ħ****K*** and the energy *E* that is explicitly displayed in Eqs. (14) and (15). This turns out to be important for the rest of the paper, where we use the many-body *T* matrix to arrive at an accurate (selfconsistent) theory of interacting quantum gases.

## 3. Fermi Gases

We start our discussion of the interacting quantum gases with the Fermi case. We will only consider spin-polarized atomic gases in which two hyperfine states are almost equally populated. For convenience, these two hyperfine states will be denoted by |↑〉 and |↓〉, respectively. The reason why we do not discuss doubly spin-polarized gases, is that in such gases the atoms can only scatter via *p*-waves due to the Pauli principle. As a result the (thermalizing) elastic collisions are dominated by the long-range magnetic dipole-dipole interaction *V*^d^ and the spatial degrees of freedom only equilibrate on a timescale that is comparable to the lifetime of the gas, i.e., *τ*_el_≃*τ*_inel_. Furthermore, if one is able to cool the system by other means, the gas basically behaves as an ideal Fermi gas on timescales short compared to the lifetime and interaction effects are negligible ([cf. Eq. (16)]).

### 3.1 The Case *a* > 0

We have argued that to account for all two-body *s*-wave scattering processes we must use the interaction

$$\begin{array}{c} H_{\mathrm{int}}=\frac{1}{V}\sum_{\mathrm{kk}\prime q}T_{↑,↓}^{MB}\left(0,0,k+k\prime ;\hbar^{2}{\left(k-k\prime \right)}^{2}/4m\right)\\ a_{k+q,↑}^{†}a_{k\prime -q,↓}^{†}a_{k\prime ,↓}a_{k,↑}. \end{array}$$ (17)

However, this is not completely true because this interaction induces on average also a change in the Hamiltonian *H*_0_, due to the fact that the occupation numbers $N_{k,\alpha }=\langle a_{k,\alpha }^{†}a_{k,\alpha }\rangle$ do not vanish. This so-called mean-field correction is clearly given by

$$\begin{array}{c} \Delta H_{0}=\sum_{\alpha }\sum_{k}a_{k,\alpha }^{†}a_{k\prime ,\alpha }\{\frac{1}{V}\sum_{\alpha \prime \neq \alpha }\sum_{k\prime }\;\\ T_{↑,↓}^{MB}\left(0,0,k+k\prime ;\hbar^{2}{\left(k-k\prime \right)}^{2}/4m\right)N_{k\prime ,\alpha \prime }\}, \end{array}$$ (18)

and, denoting the density of atoms in the spin state |*α*〉 by *n*_α_, is well approximated by

$$\begin{array}{c} \Delta H_{0}=\sum_{\alpha }\sum_{k}a_{k,\alpha }^{†}a_{k,\alpha }\\ \left\{\sum_{\alpha \prime \neq \alpha }T^{2B}\left(0,0;0\right)n_{\alpha \prime }\right\}, \end{array}$$ (19)

since the dominant contributions to the integration over the momentum *ħ****k***′ come from the region near the Fermi momentum $\hbar {k\prime }_{F,\alpha \prime }\equiv \sqrt{2m\left(\mu_{\alpha \prime }-\varepsilon_{\alpha \prime }\right)}$ (or from the thermal momentum *ħ*/*Λ* if the gas is not degenerate) where the many-body *T* matrix is almost equal to the two-body *T* matrix. The change in *H*_0_ can, therefore, be absorbed in a suitable redefinition of the chemical potentials. Introducing

$$\mu_{\alpha }^{\prime }=\mu_{\alpha }-\sum_{\alpha \prime \neq \alpha }T^{2B}\left(0,0;0\right)\;n_{\alpha \prime },$$ (20)

the correct effective Hamiltonian for the spin-polarized Fermi gases finally becomes

$$\begin{array}{c} H^{\text{eff}}=\sum_{\alpha }\sum_{k}\left(\varepsilon_{k,\alpha }-\mu_{\alpha }^{\prime }\right)a_{k\alpha }^{†},a_{k,\alpha }-VT^{2B}\left(0,0;0\right)n_{↑}n_{↓}\\ +\frac{1}{V}\sum_{\mathrm{kk}\prime q}T_{↓,↑}^{MB}\left(0,0,k+k\prime ;\hbar^{2}{\left(k-k\prime \right)}^{2}/4m\right)\\ a_{k+q,↑}^{†}a_{k\prime +q,↓}^{†}a_{k\prime ,↓}a_{k\prime ,↑}, \end{array}$$ (21)

where we have subtracted the constant *VT*^2^*^B^*(***0***,***0***;0) *n*_↑_*n*_↓_ to avoid a double counting of the effects of the interaction that have already been included in the redefinition of the chemical potentials. Notice that we have implicitly assumed that the substitution $\mu_{\alpha }\to \mu_{\alpha }^{\prime }$ is carried out also in the occupation numbers of Eq. (15), since this makes the calculation of the many-body *T* matrix self consistent.

From this effective Hamiltonian we conclude that the spin-polarized gases are so-called Fermi liquids [9]. In particular, the elementary excitations (or quasiparticles) of the gas have a dispersion relation given by $\varepsilon_{k,\alpha }-\mu_{\alpha }^{\prime }$ and scatter off each other with an amplitude given by the many-body *T* matrix. As a result the density of atoms in the two spin states is determined by

$$n_{\alpha }=\frac{1}{V}\sum_{k}N_{k\alpha }$$ (22)

with the occupation numbers *N_kα_* equal to the Fermi distribution function (e*^βx^* + 1)^−1^ evaluated at $\varepsilon_{k,\alpha }-\mu_{\alpha }^{\prime }$. Moreover, the pressure of the gas is simply

$$\begin{array}{c} p=\frac{k_{B}T}{V}\sum_{\alpha }\sum_{k}\ln\left(1+e^{-\beta \left(\varepsilon_{k,\alpha }-\mu_{\alpha }^{\prime }\right)}\right)\\ +\frac{4\pi a\hbar^{2}}{m}n_{↑}n_{↓}\equiv \sum_{\alpha }p_{\alpha }+p_{\mathrm{int}}. \end{array}$$ (23)

For a positive scattering length *a*, the interaction between the atoms is effectively repulsive and the second term in the right-hand side is positive. Interestingly enough, this does not imply that the gas is always mechanically stable. Indeed, using the hydrodynamic equations ∂*n*_α_/∂*t* = −∇ · ***J****_α_* and ∑*_α_* ∂ ***J***_α_/∂*t* = −∇*p*/*m*, we find that the two sound modes in the gas are described by the coupled wave equations

$$\frac{\partial^{2}}{\partial t^{2}}\left[\begin{array}{c} n_{↑}\\ n_{↓} \end{array}\right]=\frac{1}{m}\left[\begin{array}{cc} \partial p_{↑}/\partial n_{↑} & \partial p_{\mathrm{int}}/\partial n_{↓}\\ \partial p_{\mathrm{int}}/\partial n_{↑} & \partial p_{↓}/\partial n_{↓} \end{array}\right]\cdot \nabla^{2}\left[\begin{array}{c} n_{↑}\\ n_{↓} \end{array}\right].$$ (24)

We thus conclude that the velocities of the sound modes are equal to the square root of the eigenvalues of the matrix in the right-hand-side of Eq. (24), and that both these eigenvalues need to be positive for the sound velocities to be real and the gas to be mechanically stable.

In the case of a nondegenerate Fermi gas we can apply Boltzmann statistics to obtain *p_α_* = *n_α_k*_B_*T*. The condition of mechanical stability then reduces to *n*_↑_ *n*_↓_*a*^6^ ⩽ (*a*/*Λ*)^4^/4 and requires rather low densities. In the more interesting case of a degenerate Fermi gas, however, we have

$$p_{\alpha }=n_{\alpha }k_{B}T\frac{1}{5}{\left(3\sqrt{\frac{\pi }{2}}n_{\alpha }\Lambda^{3}\right)}^{2/3},$$ (25)

neglecting terms of order *O*(1/(*k*_*F*,*α*_ *Λ*)^4^). The condition on the densities then becomes *n*_↑_*n*_↓_*a*^6^⩽π^2^/2304, which is much less restrictive. Note that in the mechanically unstable region, the gas is unstable against “spin-density” fluctuations and will phase-separate into two gaseous phases with the same total density *n*_↑_ + *n*_↓_ but opposite “spin” densities *n*_↑_ − *n*_↓_. This process of phase separation is known as a spinodal decomposition and the density and temperature conditions at which it first occurs, lie in our case on a so-called spinodal surface.

Apart from the first-order phase transition implied by the above instability, we do not expect any other phase transition to occur in the gas on the basis of our effective Hamiltonian. In particular, we do not expect a phase transition due to quantum degeneracy, because for that we need an attractive interaction between the quasiparticles, as will be explained shortly. In principle, however, it is possible that a positive *s*-wave scattering amplitude induces a negative *p*-wave scattering amplitude due to the Kohn-Luttinger effect [10]. In this manner a phase transition to a superfluid state might occur after all. Although it is interesting to analyze this possibility in more detail, we will not do so here since it is beyond the scope of the present paper.

### 3.2 The Case *a* < 0

The prospects for achieving a phase transition in the degenerate regime are more promising for spin-polarized Fermi gases with a negative scattering length, which actually applies to both atomic deuterium [11] and atomic ^6^Li [12]. Nevertheless, for this phase transition to be experimentally observable, it must take place in the (meta)stable region of the phase diagram where the gas is mechanically stable. Since the stability analysis of the previous section is insensitive to the sign of scattering length, this implies in the degenerate regime that the densities of the two spin states have to fulfill the requirement *n*_↑_ *n*_↓_a^6^ ⩽ π ^2^/2304. In contrast to the case of a Fermi gas with a positive scattering length, the latter condition now does not rule out the possibility of the formation of a superfluid state at sufficiently low temperatures.

The physical reason for this is that with effectively attractive interatomic interactions the gas can form a Bose condensate of Cooper pairs, in the same way as an electron gas can form a condensate of Cooper pairs in the BCS theory of superconductivity [9]. Mathematically, the instability of the gas towards the creation of Cooper pairs is signalled by the fact that the scattering amplitude diverges for two quasiparticles at the Fermi energy $\varepsilon_{F}\equiv \hbar^{2}k_{F}^{2}/2m=\left(\hbar^{2}k_{F,↑}^{2}/2m+\hbar^{2}k_{F,↓}^{2}/2m\right)/2$ with opposite momenta and “spin” [13]. Using Eqs. (14) and (15) and neglecting the small imaginary part of *Ξ* (***0***; 2*ε*_F_), we thus find that the critical temperature of the BCS transition obeys

$$\begin{array}{c} \frac{1}{T_{↑,↓}^{MB}\left(0,0,0;2\varepsilon_{F}\right)}=\frac{m}{4\pi a\hbar^{2}}+℘\int \frac{dk}{{\left(2\pi \right)}^{3}}\\ \frac{N_{↑}\left(k\right)+N_{↓}\left(k\right)}{2\varepsilon_{F}-\hbar^{2}k^{2}/m}=0, \end{array}$$ (26)

where ℘ denotes the Cauchy principal-value part of the integral.

The properties of this so-called linearized BCS gap equation are well-known from the theory of superconductivity. First, this equation only has a solution if *a* < 0. Second, the highest critical temperature for a given total density *n* is obtained if *n*_↑_ = *n*_↓_ or equivalently if $\delta \varepsilon_{F}=\left|\hbar^{2}k_{F{,}_{↑}}^{2}/2m-\hbar^{2}k_{F{,}_{↓}}^{2}/2m\right|=0$. This can be understood most easily by noting that for a nonzero value of δ*ε*_F_ there are less particles at the Fermi surface that can be paired, which suppresses the critical temperature. Third, if the densities in both spin states are equal, the gap equation can be solved analytically [14], and leads to

$$T_{c}≃\frac{5\varepsilon_{F}}{3k_{B}}\exp\left\{-\frac{\pi }{2k_{F}|a|}-1\right\}.$$ (27)

For unequal densities an analytical treatment is not feasible, but a numerical solution of Eq. (26) shows that nonzero critical temperatures are only possible if the “spin polarization” |*n*_↑_ − *n*_↓_|/(*n*_↑_ + *n*_↓_) is less than 3*k*_B_*T*_c_/2*ε*_F_ [12]. Qualitatively, this is a result of the fact that if δ*ε*_F_ is of the order of *k*_B_*T*_c_, it is no longer energetically favorable to form Cooper pairs and the gas is in a normal state even at zero temperature.

From an experimental point of view, we thus conclude that the most favorable conditions for the observation of the BCS transition in a spin-polarized Fermi gas are obtained if *n*_↑_ = *n*_↓_ = *n*/2. Under these conditions the mechanical stability of the gas at the temperatures of interest requires that *n*|*a*|^3^ ⩽ *π*/24 or equivalently that ***k***_F_|*a*| ⩽ *π*/2. For atomic deuterium the triplet scattering length is equal to *a* ≃ −6.8 *a*_0_ (*a*_0_ is the Bohr radius). At realistic densities of ~10^12^ cm^−3^ we thus find that ***k***_F_|*a*| ≃ 1.1 × 10^23^ << *π*/2 and the stability condition is easily fulfilled. However, from the exponential dependence in Eq. (27) we immediately see that the critical temperature is completely out of reach with present-day cooling techniques. Fortunately, this conclusion does not hold for atomic ^6^Li because the triplet scattering length now has the anomalously large value of *a* ≃ − 4.6 × 10^3^ *a*_0_. For the same density of atoms we thus find that ***k***_F_|*a*| ≃ 0.4 < *π*/2 and that the critical temperature is only 29 *nK*, which is rather close to the temperatures that recently have been obtained with the bosonic isotope ^7^Li [2]. Therefore, it appears that ^6^Li is a very promising candidate for the observation of the BCS transition in a weakly interacting gas.

Because of this possible application, we also want to mention briefly how we can arrive at an accurate description of the gas below the critical temperature. Although it is not difficult to treat the general situation with *n*_↑_ ≠ *n*_↓_, we concentrate here on the most important case of an equal population of the two hyperfine states. Moreover, we also only discuss the derivation of the elementary excitations of the superfluid state (which is sufficient for the most interesting equilibrium properties of the gas) and do not consider the effective interaction between these quasiparticles. Introducing the convenient notation $\mu \prime \equiv \mu_{\alpha }^{\prime }-\varepsilon_{\alpha }$ for the renormalized chemical potential (or Fermi energy *ε*_F_) of both spin states, our task is therefore reduced to including into the free Hamiltonian of the quasi particles, i.e., into

$$H_{0}^{\text{eff}}\equiv \sum_{\alpha }\sum_{k}\left(\varepsilon_{k}-\mu \prime \right)\;a_{k,\alpha }^{†}a_{k,\alpha }-VT^{2B}\left(0,0;0\right)\frac{n^{2}}{4},$$ (28)

the effect of the Cooper pairs.

To achieve this, we must realize that BCS theory is roughly speaking the theory of a Bose-Einstein condensation of Cooper pairs. Therefore, the order parameter of the phase transition is the expectation value of the annihilation operator for a pair of atoms. (See Sec. 4 for a treatment of Bose-Einstein condensation in an atomic Bose gas.) As a result, we do not only have nonvanishing average occupation numbers $\langle a_{k,\alpha }^{†}a_{k,\alpha \prime }\rangle$ but also nonvanishing values for 〈*a*_*k*,*α*_ *a*_−*k*,*α*__′_〉. Note that in our case we are dealing with a pairing between atoms with different spin states and the latter average is only nonzero for *α* ≠ *α*′. To see what the influence of the anomalous averages 〈*a*_*k*,↑_ *a*_−*k*,↓_〉 is, we briefly return to the Hamiltonian of Eq. (1). Taking for the sake of clarity *V_q_* = *V_0_*, the lowest order average correction on the Hamiltonian *H*_0_ due to the interaction now becomes [cf. Eq. (19)]

$$\begin{array}{c} \Delta H_{0}=\sum_{\alpha }\sum_{k}a_{k,\alpha }^{†}a_{k,\alpha }\left\{\sum_{\alpha \prime \neq \alpha }V_{0}n_{\alpha \prime }\right\}\\ +\sum_{k}\left\{\Delta_{0}a_{k,↑}^{†}+\Delta_{0}^{*}a_{-k,↓}a_{k,↑}\right\}, \end{array}$$ (29)

if we define the BCS gap parameter *Δ*_0_ by

$$\Delta_{0}\equiv \frac{1}{V}\sum_{k}V_{0}\langle a_{-k,↓}a_{k,↑}\rangle .$$ (30)

From our previous discussion of the normal state of the gas we know that we can to a good approximation account for all two-body processes by replacing in Eq. (29) the potential *V*_0_ by *T*^2B^(***0***, ***0***; 0). It is important, however, that we do not perform this substitution in Eq. (30) because the BCS theory is already going to sum all the other relevant two-body processes for us [15].

We thus arrive at the conclusion that the free Hamiltonian of the quasiparticles, including the effects of a condensate of Cooper pairs, is given by

$$\begin{array}{c} H_{0}^{\text{eff}}=\sum_{\alpha }\sum_{k}\left(\varepsilon_{k}-\mu \prime \right)\;a_{k,\alpha }^{†}a_{k,\alpha }+\sum_{k}\;\\ \left\{\Delta_{0}a_{k,↑}^{†}a_{-k{,}_{↓}}^{†}+\Delta_{0}^{*}a_{-k,↓}a_{k,↑}\right\}\\ -V\frac{{\left|\Delta_{0}\right|}^{2}}{V_{0}}-VT^{2B}\left(0,0;0\right)\frac{n^{2}}{4}, \end{array}$$ (31)

where we have again subtracted a constant term to avoid a double counting of the interaction effects. Being quadratic in the creation and annihilation operators, this Hamiltonian can be diagonalized by means of a Bogoliubov transformation, and we find that the Bogoliubov quasiparticles have an energy dispersion $\hbar \omega_{k}=\sqrt{{\left(\varepsilon_{k}-\mu \prime \right)}^{2}+{\left|\Delta_{0}\right|}^{2}}$ which for *n*_↑_ = *n*_↓_ is independent of their “spin.” Moreover, after this diagonalisation the average density *n* and order parameter *Δ*_0_ are easily calculated and result in the equation of state

$$n=\frac{1}{V}\sum_{\alpha }\sum_{k}\left\{\frac{\varepsilon_{k}-\mu \prime }{\hbar \omega_{k}}N_{k,\alpha }+\frac{\hbar \omega_{k}-\varepsilon_{k}+\mu \prime }{2\hbar \omega_{k}}\right\}$$ (32)

and the BCS gap equation

$$\frac{1}{V_{0}}+\frac{1}{V}\sum_{k}\frac{1-N_{k,↑}-N_{k,↓}}{2\hbar \omega_{k}}=0,$$ (33)

respectively. Here, the average occupation numbers of the quasiparticles are again denoted by *N*_*k*,*α*_, but they are now equal to the Fermi distribution function evaluated at *ħ*ω*_k_* and are in fact independent of *α*.

The equation of state and the BCS gap equation are, at a given density and temperature, two equations for the two unknown quantities *μ*′ and |*Δ*_0_|. However, before we can actually solve these equations we first have to resolve the ultraviolet divergence in the gap equation. This divergence is a consequence of the fact that we have neglected the momentum dependence of the potential and used *V_q_* = *V_0_*. Making use of the same approximation in the Lippman-Schwinger equation Eq. (7) and of the fact that the Fermi energy $\mu \prime =\hbar^{2}k_{F}^{2}/2m$ is always much smaller than $\hbar^{2}/mr_{V}^{2}$, we find that the two-body *T* matrix obeys

$$\frac{1}{T^{2B}\left(0,0:0\right)}≃\frac{1}{T^{2B}\left(0,0:2\mu \prime \right)}=\frac{1}{V_{0}}+\frac{1}{V}\sum_{k}\frac{1}{2\left(\varepsilon_{k}-\mu \prime \right)}$$ (34)

in this case. We thus observe that the divergence in the BCS gap equation can be cancelled by a renormalization of 1/*V_0_* to 1/*T*^2B^***0*** (***0***,***0***;0) = *m*/4*paħ*^2^. In this manner we then obtain the following gap equation

$$\frac{m}{4\pi a\hbar^{2}}+\frac{1}{V}\sum_{k}\left\{\frac{1-N_{k,↑}-N_{k,↓}}{2\hbar \omega_{k}}-\frac{1}{2\left(\varepsilon_{k}-\mu \prime \right)}\right\}=0,$$ (35)

which is free of divergences. Furthermore, taking the limit *Δ*_0_ → 0 to obtain an equation for the critical temperature *T*_c_, we exactly reproduce (after taking also the thermodynamic limit and substituting *μ*′ = *ε*_F_) the result of Eq. (26) which shows the consistency of our approach and, in particular, also that the BCS theory in essence indeed sums all two-body scattering processes.

## 4. Bose Gases

We now turn our attention to the atomic Bose gases, which have caused so much excitement in the atomic physics community after the experimental observation of Bose-Einstein condensation in atomic ^87^Rb, ^7^Li and ^23^Na vapours. As in these experiments, we consider here only the doubly spin-polarized case. This implies that there is only one spin state in the problem and we will, therefore, in this section completely suppress the spin indices in our notation.

### 4.1 The Case *a* > 0

Again, we first consider a gas with an effectively repulsive triplet interaction, which applies for example to atomic hydrogen [6], atomic ^87^Rb [16] and atomic ^23^Na [17]. The appropriate effective Hamiltonian for the doubly spin-polarized atomic Bose gases is, in analogy with the discussion in Sec. 3.1, given by

$$\begin{array}{c} H^{\text{eff}}=\sum_{k}\left(\varepsilon_{k}-\mu \prime \right)a_{k}^{†}a_{k}-VT^{2B}\left(0,0;0\right)n^{2}\\ +\frac{1}{2V}\sum_{\mathrm{kk}\prime q}T^{MB}\left(0,0,k+k\prime ;\hbar^{2}{\left(k-k\prime \right)}^{2}/4m\right)\\ a_{k+q}^{†}a_{k\prime -q}^{†}a_{k\prime }a_{k}, \end{array}$$ (36)

with a renormalized chemical potential *μ*′ equal to

$$\mu \prime =\mu -2nT^{2B}\left(0,0;0\right).$$ (37)

Looking at this hamiltonian we immediately see that the density of atoms is determined by

$$n=\frac{1}{V}\sum_{k}N_{k}$$ (38)

where the average occupation numbers *N_k_* are now equal to the Bose distribution function (e*^β x^* − 1)^−1^ evaluated at *ε_k_* − *μ*′. In addition, the pressure of the gas is

$$p=-\frac{k_{B}T}{V}\sum_{k}\ln\left(1-e^{-\beta \left(\varepsilon_{k}-\mu \prime \right)}\right)+\frac{4\pi a\hbar^{2}}{m}n^{2},$$ (39)

which for a gas with a positive scattering length does not show a mechanical instability, i.e., d*p*/d*n* is always greater than zero for densities such that *nr_V_*^3^<< 1 and our theory is applicable.

On the basis of Eq. (38) we thus expect the gas to Bose condense at the same critical temperature

$$T_{c}=T_{0}\equiv \frac{2\pi \hbar^{2}}{mk_{B}}\left(\frac{n}{\zeta \left(3/2\right)}\right)2/3$$ (40)

as the ideal Bose gas. For an interacting Bose gas with positive scattering length, the order parameter corresponding to this phase transition is the expectation value of the annihilation operator for an atom [18]. If we also want to describe the gas below the critical temperature, we need to consider the influence of a nonvanishing average 〈*a_0_*〉 on the effective Hamiltonian. Focussing again on the derivation of the elementary excitations in the superfluid state, this is most easily achieved by first carrying out the substitution *a*_*k*_ → δ_*k*,*0*_ 〈*a_0_*〉 + *a_k_* in the Hamiltonian *H*^eff^, and then keeping only all terms that are of first or second order in the creation and annihilation operators. In this manner we find in a good approximation that

$$\begin{array}{c} H_{0}^{\text{eff}}=-V(T^{2B}\left(0,0:0\right){\left(n\prime \right)}^{2}+2T^{MB}\left(0,0,0;0\right)n\prime n_{0}\\ +\frac{1}{2}T^{MB}\left(0,0,0;0\right)n_{0}^{2})\\ +\sqrt{Vn_{0}}\left(-\mu \prime +n_{0}T^{MB}\left(0,0,0;0\right)\right)\left(a_{0}+a_{0}^{†}\right)\\ +\sum_{k}\left(\varepsilon_{k}-\mu \prime \right)a_{k}^{†}a_{k}+\sum_{k}\{\left(2n_{0}T^{MB}\left(0,0,0;0\right)\right)a_{k}^{†}a_{k}\\ +\frac{1}{2}n_{0}\;T^{MB}\left(0,0,0;0\right)\left(a_{k}^{†}a_{-k}^{†}+a_{-k}a_{k}\right)\}, \end{array}$$ (41)

if we choose 〈*a_0_*〉 to be real and introduce the condensate density *n*_0_ ≡ 〈*a_0_*〉^2^/*V* and the density of noncondensed atoms *n*′ = *n* − *n*_0_. The most difficult part of the calculation is again to avoid double countings of the interaction effects. Besides the subtractions in the first term in the right-hand-side, this also requires that the relation between *μ* and *μ*′ is changed into *μ* = 2*n*′ *T*^2^*^B^*(***0***, ***0***; 0) + *μ*′, which reduces to Eq. (37) if the condensate density vanishes.

To proceed, we first note that if we want 〈*a_0_*〉 to be the total expectation value of the original operator *a_0_*, we must require that the expectation value of the new operator *a_0_* (i.e., after the substitution) disappears. This is achieved by putting *μ*′ = *n*_0_ *T^MB^*(***0***, ***0***, ***0***; 0), because then the terms in the hamiltonian linear in the creation and annihilation operators vanish. As a result, the chemical potential is determined by

$$\mu =2\left(n-n_{0}\right)T^{2B}\left(0,0;0\right)+n_{0}T^{MB}\left(0,0,0;0\right),$$ (42)

which for the approximation that we are using is just the famous Hugenholtz-Pines relation [19]. Because our theory obeys this relation, we expect that the dispersion relation *ħ*ω*_k_* of the quasiparticles is linear at long wavelengths. This indeed turns out to be the case, since a diagonalization of the above hamiltonian by means of a Bogoliubov transformation shows that $\hbar \omega_{k}=\surd \bar{{\varepsilon^{2}}_{k}+2n_{0}T^{MB}\left(0,0,0;0\right)\varepsilon_{k}}$ [20] and that

$$\begin{array}{c} H_{0}^{\text{eff}}=\sum_{k}\hbar \omega_{k}b_{k}^{†}b_{k}\\ -V(T^{2B}\left(0,0;0\right){\left(n\prime \right)}^{2}+2T^{MB}\left(0,0,0;0\right)n\prime n_{0}\\ +\frac{1}{2}T^{MB}\left(0,0,0;0\right){n_{0}}^{2}), \end{array}$$ (43)

where $b_{k}^{†}$ and *b_k_* denote the creation and annihilation operators for the Bogoliubov quasiparticles, and we have been neglecting between the brackets additional terms that are a factor of order $O\left(\sqrt{n_{0}a^{3}}\right)$ smaller [18]. Moreover, using this Bogoliubov transformation we can now obtain also for the superfluid state the pressure

$$\begin{array}{c} p=-\frac{k_{B}T}{V}\sum_{k}\ln\left(1-e^{-\beta \hbar \omega_{k}}\right)\\ +T^{2B}\left(0,0;0\right){\left(n\prime \right)}^{2}+2T\left(0,0,0;0\right)n\prime n_{0}\\ +\frac{1}{2}T^{MB}\left(0,0,0;0\right){n_{0}}^{2} \end{array}$$ (44)

and the equation of state

$$\begin{array}{c} n=n_{0}+\sum_{k}\{\frac{\varepsilon_{k}+n_{0}T^{MB}\left(0,0,0;0\right)}{\hbar \omega_{k}}N_{k}\\ +\frac{\varepsilon_{k}+n_{0}T^{MB}\left(0,0,0;0\right)-\hbar \omega_{k}}{2\hbar \omega_{k}}\}, \end{array}$$ (45)

which determines the condensate density at a fixed density and temperature. Note that in the equation of state the average occupation numbers of the quasiparticles *N_k_* are equal to the Bose distribution function evaluated at *ħω_k_*.

This almost completes our discussion of the interacting Bose gas with a positive scattering length. However, there remains one point that we need to address, namely that our theory is not yet self consistent. This is because we have not yet mentioned how the many-body *T* matrix is determined below the critical temperature *T*_c_. In general, this is a complicated problem due to the presence of infrared divergences in the theory of a dilute Bose gas [21]. At very low temperatures such that *na****Λ***^2^ ≫ 1 the treatment of these divergences requires more advanced methods then the ones we are using here. Therefore, we consider only the opposite limit *na****Λ***^2^ ≪ 1, which is in fact the most relevant one for experiments. In this regime the average kinetic energy ***k***_B_*T* of the atoms is much larger than the average interaction energy 4*πanħ*^2^/*m* and the Bogoliubov dispersion is for all practical purposes well approximated by *ħω_k_*. *ε_k_* + *n*_0_ *T^MB^*(***0***, ***0***, ***0***; 0) = *ε_k_* + *μ′*. In Eq. (41) this means that we can neglect the anomalous terms proportional to $a_{k}^{†}a_{-k}^{†}$ or *a*_−_*_k_a_k_*. Hence, we now basically deal with the Hamiltonian $\sum_{k}\left(\varepsilon_{k}+\mu \prime \right)a_{k}^{†}a_{k}$ and the many-body *T* matrix is again just given by Eqs. (14) and (15). Finally, we have to deal with one last subtlety. In the way we have defined the many-body *T* matrix, it describes the scattering of quasiparticles. However, we are dealing with a Bose condensate of particles and we actually need their scattering amplitude. In our case, fortunately, this only leads to a slight shift *E* →*E*−2*μ′* in the energy of the many-body *T* matrix. We thus arrive at a fully self-consistent theory of the superfluid phase in the regime *naΛ*^2^ ≪ 1, if in all formulas below Eq. (40) *T^MB^*(***0***, ***0***, ***0***; 0) is replaced by the many-body *T* matrix *T*^MB^(***0***, ***0***, 0;−2*μ′*).

The transition matrix *T*^MB^(***0***, ***0***, 0; −2*μ′*) is almost equal to *T*^2B^(***0***, ***0***; 0) = 4*π*a*ħ*^2^/*m* for most temperatures below *T*_c_, but decreases to zero for temperatures near the critical temperature due to the fact that *Ξ* (***0***; −2*μ′*) diverges in the limit *μ′* →0. Therefore, our self-consistent approach reduces to the conventional Bogoliubov (or Popov) theory of a weakly interacting Bose gas for most temperatures. However, near the critical temperature it resolves a long-standing problem of the Bogoliubov theory, which actually predicts a first-order phase transition [22] instead of the second-order phase transition expected from the theory of critical phenomena. Of course, using the many-body *T* matrix instead of the two-body *T* matrix does not include all the critical fluctuations that are of importance near the critical temperature. Nevertheless, using the many-body *T* matrix we incorporate, at least qualitatively correctly, the fact (known from renormalization group theory [23]) that the interaction between the condensate particles should vanish exactly at the critical temperature. It is rather interesting that this can already be achieved by including only all two-body scattering processes [24].

### 4.2 The Case *a* < 0

We now turn to the physics of a Bose gas with effectively attractive interactions, which is realized for instance in atomic ^7^Li [25] and atomic ^133^Cs [26]. This is an interesting topic because a thoughtless application of the theory of Sec. 4.1 would result in a dispersion of the Bogoliubov quasiparticles that is equal to $\hbar \omega_{k}=\sqrt{\varepsilon_{k}^{2}}-\left(8\pi |a|n_{0}\hbar^{2}/m\right)\varepsilon_{k}$ and therefore purely imaginary at long wavelengths. From this simple argument we can thus already conclude that Bose-Einstein condensation in the canonical sense, i.e., with the order parameter 〈*a_0_*〉, will not occur if the scattering length is negative [27]. However, from the experience with fermionic gases gained in Sec. 3.2, we might expect a BCS-like phase transition to take place instead. This would be signalled in Eq. (36) by a divergence of the scattering amplitude of two quasiparticles at energy *μ*′ and with opposite momenta, or by [cf. Eq. (26)]

$$\begin{array}{c} \frac{1}{T^{MB}\left(0,0,0;2\mu^{\prime }\right)}=\\ \frac{1}{T^{2B}\left(0,0;0\right)}-\int \frac{dk}{{\left(2\pi \right)}^{3}}\frac{N\left(k\right)}{\mu \prime -\hbar^{2}k^{2}/2m}=0 \end{array}$$ (46)

with of course *N*(***k***) the Bose distribution function evaluated at *ħ* ^2^***k***^2^/2*m* − *μ*′. Analyzing this condition, we find that the phase transition indeed occurs at a temperature *T*_BCS_ = *T*_0_(1 + *O* (|*a*|/*Λ*0)), which is only slightly above the critical temperature of the ideal Bose gas.

Following the same procedure as in Sec. 3.2, we can easily show that below this critical temperature the equation of state becomes

$$n\frac{1}{V}\sum_{k}\left\{\frac{\varepsilon_{k}-\mu \prime }{\hbar \omega_{k}}N_{k}+\frac{\varepsilon_{k}-\mu \prime -\hbar \omega_{k}}{2\hbar \omega_{k}}\right\}$$ (47)

and that the BCS gap equation is well approximated by

$$\frac{m}{4\pi a\hbar^{2}}+\frac{1}{V}\sum_{k}\frac{N_{k}}{\hbar \omega_{k}}=0.$$ (48)

Here the energy of the Bogoliubov quasiparticles is $\hbar \omega_{k}=\sqrt{{\left(\varepsilon_{k}-\mu \prime \right)}^{2}-{\left|\Delta_{0}\right|}^{2}}$ and their average occupation numbers *N_k_* are equal to the Bose distribution function evaluated at this energy. These equations again determine *μ*′ and |*Δ*_0_| at a fixed density and temperature. Notice that in contrast with the fermion case, the dispersion *ħ*ω*_k_* has a minus sign in front of |*Δ*_0_|^2^. This has important consequences because it implies that if we lower the temperature at a fixed density, both |*Δ*_0_|^2^ and *μ*′ increase in such a manner that the gap in the Bogoliubov dispersion decreases. Hence, at a second temperature *T*_BEC_ < *T*_BCS_, the gap closes and the number of particles in the zero-momentum state diverges, which signals a Bose-Einstein condensation. Below that second critical temperature we always have |*Δ*_0_|= −*μ*′ and the dispersion *ħv_k_* becomes equal to $\sqrt{\varepsilon_{k}^{2}-2\mu^{\prime }\varepsilon_{k}}$ which, interestingly enough, is exactly the same Bogoliubov dispersion as for a Bose-condensed gas with positive scattering length. Moreover, Eqs. (47) and (48) turn into

$$n=n_{0}+\frac{1}{V}\sum_{k\neq 0}\left\{\frac{\varepsilon_{k}-\mu \prime }{\hbar \omega_{k}}N_{k}+\frac{\varepsilon_{k}-\mu \prime -\hbar \omega_{k}}{2\hbar \omega_{k}}\right\}$$ (49)

and

$$\frac{m}{4\pi a\hbar^{2}}-\frac{n_{0}}{\mu \prime }+\frac{1}{V}\sum_{k\neq 0}\frac{N_{k}}{\hbar \omega_{k}}=0,$$ (50)

determining now *μ*′ and the condensate density *n*_0_. It is important to mention here that the possibility of a Bose-Einstein condensation in a gas with effectively attractive interactions, in the way that we have just seen, does not contradict the argument against Bose-Einstein condensation presented in the beginning of this section. The reason is that in that argument we assumed that a Bose-Einstein condensation would be associated with the order parameter 〈*a_0_*〉 and did not consider the possibility that it would be associated with the condensate density *n*_0_, as in the ideal Bose gas [27].

It thus appears that a Bose gas with a negative scattering length offers the exciting possibility of observing both a BCS transition and a Bose-Einstein condensation. However, we have up to now not considered the mechanical stability of the gas in these two superfluid phases. This is clearly of the utmost importance, because Eq. (39) shows that for a negative scattering length, d*p*/d*n* does not always have to be positive. Indeed, calculating the spinodal line from d*p*/d*n* = 0, we actually find the unfortunate result that the gas is always unstable at *T*_BCS_. We are thus forced to conclude that the two second-order phase transitions that we have found above are always preempted by a first-order gas-liquid or gas-solid transition and are, therefore, in a homogeneous system unobservable.

## 5. Discussion and Outlook

In this paper we have presented a unified picture of weakly interacting atomic gases, focussing on the differences that arise due to the statistics of the atoms and due to the interatomic interaction being either effectively repulsive or attractive. However, we have restricted our discussion to the homogeneous case, whereas the experiments are up till now always performed in an inhomogeneous magnetic trap. Fortunately, the experiments are also always performed at temperatures such that *k*_B_*T* >> *ħ*ω, where *ħ*ω is the energy splitting of the (harmonic oscillator) trapping potential. Under these conditions we are generally justified in using a local-density approximation, which means that we consider the gas as homogeneous in each point in space. In this sense the theory presented above can also be applied to an inhomogeneous situation and, in first instance, we do not anticipate any qualitative differences. Nevertheless, for quantitative predictions of, for example, the critical temperature and the density profile in the trap, it is of course essential to include the effect of the trapping potential.

Moreover, for a Bose gas with negative scattering length there is also a qualitative difference. In Sec. 4.2 we argued that for a homogeneous gas a Bose condensate is always unstable. However, first Hulet [28] and subsequently Ruprecht *et al.* [29] have argued that in an inhomogeneous situation one can have a metastable condensate if the number of particles is sufficiently small. This was recently put on a more firm theoretical basis by Kagan *et al.* [30] and by one of us [31]. In particular, the last author has shown by a calculation of the decay rate of the condensate due to thermal fluctuations, that a long-lived metastable condensate can exist for temperatures sufficiently close to the critical temperature. Although this result appears to explain the recent observation of Bose-Einstein condensation in atomic ^7^Li, our theoretical understanding of these experiments is still incomplete. The most important problem that remains to be solved is the stability of the gas just above the critical temperature. Clearly, on the basis of a local-density approximation we would conclude, as in Sec. 4.2, that the gas is unstable, and that also in an inhomogeneous situation Bose-Einstein condensation is unobservable. However, the local-density approximation is invalid near the critical temperature due to the large value of the (homogeneous) correlation length. A thorough discussion of this point, therefore, requires a theory that goes beyond this approximation and is evidently of great interest in view of the Rice experiments with ^7^Li [2].

Also in the case of a Bose gas with positive scattering length, interesting theoretical problems remain. One of them is the correct treatment of the infrared divergences in the regime *naΛ*^2^ ≫ 1, which, due to the effectiveness of evaporative cooling techniques, is also anticipated to be experimentally accessible. As already mentioned, we presumably need more advanced methods than discussed here to accurately perform a resummation of the divergent contributions to the thermodynamic quantities of interest. Indeed, we are presently carrying out a renor-malization group study of the interacting Bose gas in this regime, because by this method infrared divergences are automatically avoided. Moreover, a resolution of these divergences is also of importance for the theory of two-dimensional atomic Bose gases, such as doubly spin-polarized atomic hydrogen adsorbed on su-perfluid ^4^He films. The reason for this is that in a two-dimensional geometry, the divergences are so important that they actually prevent the possibility of a Bose condensate (in the thermodynamic limit). Nevertheless, the gas is expected to undergo a phase transition to a super-fluid state at sufficiently low temperatures. In our opinion, formulating an accurate microscopic theory [32] for this so-called Kosterlitz-Thouless transition is an important challenge for the future. Finally, we would also like to mention the possibility of studying mixtures of Fermionic and Bosonic atomic gases. One exciting possibility is a mixture of doubly spin-polarized atomic ^6^Li and ^7^Li. In this manner one would create the dilute analogue of ^3^He and ^4^He mixtures. Such a mixture of doubly spin-polarized atomic ^6^Li and ^7^Li is rather interesting because, as we have seen in Sec. 3, the ^6^Li atoms essentially do not interact with each other but only with the ^7^Li atoms. However, due to the latter interaction they can now also be evaporatively cooled on a time scale which is much smaller than their lifetime. It therefore seems worthwhile to extend the methods discussed in this paper to this system, and in particular to study Bose-Einstein condensation in the presence of an essentially ideal Fermi gas.

It is clear from these examples, and from many others that we unfortunately are not able to discuss here, that we are heading towards an exciting future, in which no doubt many surprises will await us. We hope that this paper will be of some use to people that intend to contribute to this rapidly developing area of atomic physics.
